# Hippocampal Neurodegenerative Pathology in Post-stroke Dementia Compared to Other Dementias and Aging Controls

**DOI:** 10.3389/fnins.2017.00717

**Published:** 2017-12-19

**Authors:** Rufus O. Akinyemi, Louise M. Allan, Arthur Oakley, Rajesh N. Kalaria

**Affiliations:** ^1^Neuroscience and Ageing Research Unit, Institute for Advanced Medical Research and Training, College of Medicine, University of Ibadan, Oyo, Nigeria; ^2^Neurovascular Research Group, Institute of Neuroscience, Newcastle University, Newcastle upon Tyne, United Kingdom

**Keywords:** Alzheimer's disease, cerebrovascular disease, mixed dementia, post-stroke dementia, stroke, vascular dementia

## Abstract

Neuroimaging evidence from older stroke survivors in Nigeria and Northeast England showed medial temporal lobe atrophy (MTLA) to be independently associated with post-stroke cognitive impairment and dementia. Given the hypothesis ascribing MTLA to neurodegenerative processes, we assessed Alzheimer pathology in the hippocampal formation and entorhinal cortex of autopsied brains from of post-stroke demented and non-demented subjects in comparison with controls and other dementias. We quantified markers of amyloid β (total Aβ, Aβ-40, Aβ-42, and soluble Aβ) and hyperphosphorylated tau in the hippocampal formation and entorhinal cortex of 94 subjects consisting of normal controls (*n* = 12), vascular dementia, VaD (17), post-stroke demented, PSD (*n* = 15), and post-stroke non-demented, PSND (*n* = 23), Alzheimer's disease, AD (*n* = 14), and mixed AD and vascular dementia, AD_VAD (*n* = 13) using immunohistochemical techniques. We found differential expression of amyloid and tau across the disease groups, and across hippocampal sub-regions. Among amyloid markers, the pattern of Aβ-42 immunoreactivity was similar to that of total Aβ. Tau immunoreactivity showed highest expression in the AD and mixed AD and vascular dementia, AD_VaD, which was higher than in control, post - stroke and VaD groups (*p* < 0.05). *APOE* ε4 allele positivity was associated with higher expression of amyloid and tau pathology in the subiculum and entorhinal cortex of post-stroke cases (*p* < 0.05). Comparison between PSND and PSD revealed higher total Aβ immunoreactivity in PSND compared to PSD in the CA1, subiculum and entorhinal cortex (*p* < 0.05) but no differences between PSND and PSD in Aβ-42, Aβ-40, soluble Aβ or tau immunoreactivities (*p* > 0.05). Correlation of MMSE and CAMCOG scores with AD pathological measures showed lack of correlation with amyloid species although tau immunoreactivity demonstrated correlation with memory scores (*p* < 0.05). Our findings suggest hippocampal AD pathology does not necessarily differ between demented and non-demented post-stroke subjects. The dissociation of cognitive performance with hippocampal AD pathological burden suggests more dominant roles for non-Alzheimer neurodegenerative and / or other non-neurodegenerative substrates for dementia following stroke.

## Introduction

Stroke accounts for half of the global burden of neurological disorders while remaining the most common cause of acquired disability, and a common cause of cognitive impairment and dementia (Kalaria et al., 2016; Writing Group Members et al., 2016; GBD 2015 Neurological Disorders Collaborator Group, 2017). Magnetic resonance imaging studies in a cohort of older African stroke survivors participating in the Cognitive Function After STroke (CogFAST)—Nigeria Study showed that medial temporal lobe atrophy (MTLA) was an independent predictor of post-stroke cognitive impairment and dementia (Akinyemi et al., 2014, 2015). These findings were in concordance with previous findings of MTLA significantly predicting progression to cognitive impairment, dementia and death in the longitudinal CogFAST Newcastle Study (Firbank et al., 2007, 2012) while pathological studies unmasked hippocampal neuronal atrophy as an important neuropathological substrate of post-stroke dementia (PSD) in the Newcastle cohort (Gemmell et al., 2012).

The link between cerebrovascular disease, neurodegeneration and cognition has long been debated (de la Torre and Mussivand, 1993; Kalaria et al., 1993b). Evidence for this link has been provided by experimental models (Kalaria et al., 1993a; Kitaguchi et al., 2009) and from epidemiological studies (Schneider et al., 2004; Petrovitch et al., 2005). Accumulation of Alzheimer pathology in primary vascular brain disorders occurs largely as a result of shared mechanisms of neurovascular unit dysfunction (Iadecola, 2004; Kalaria et al., 2012). Experimental evidence from animal studies has shown that amyloid production may be exacerbated by cerebral hypoxia/ischaemia (Kalaria et al., 1993a; Whitehead et al., 2005).

In a large post-mortem study, Aho and colleagues using immunohistochemistry found no significant increase in amyloid load in subjects with cerebrovascular disease (CVD) compared to controls although there was a trend of increased deposition of Aβ-42 over Aβ-40 (Aho et al., 2006). In this context a post-mortem study of aging controls and subjects with vascular dementia (VaD), reported increased accumulation of total guanidine HCl extractable Aβ-42 peptides (over Aβ-40) by Enzyme Linked Immunosorbent Assay (ELISA) in the temporal cortex in the oldest VaD subjects as well as in older aged controls (Lewis et al., 2006) although these increases were not evident by immunohistochemistry.

Cross-sectional studies examining the relationship of Aβ with cognitive function have also yielded conflicting results. Whereas, some studies have reported significant correlation between metrics of AD pathology and cognitive performance (Bennett et al., 2006; Mormino et al., 2009) others have reported dissociation between Alzheimer pathological load and cognitive status especially in subjects with presumed high cognitive reserve, mixed pathologies or non-AD subjects (Mufson et al., 1999; Aizenstein et al., 2008; Stern, 2009). Similarly, the morphological variants and anatomical localization of the AD pathology may be important. Neuritic plaques and neurofibrillary tangles have stronger impact on cognition than diffuse plaques, and pathologies in the neocortical region exert more influence than those in the allocortical regions (Nelson et al., 2009, 2012).

The hippocampus plays a very strategic role in the neurobiology of memory, being involved in the hierarchical spread of amyloid and tau (neurofibrillary tangles) (Braak and Braak, 1991; Thal et al., 2002b). Whereas, amyloid is deposited in the hippocampus in late stages, tau deposition occurs quite early within the hippocampal formation during their natural histories, and both deposits appear to relate to the connections of the hippocampal circuitry (Lace et al., 2009).

Given the hypothesis ascribing MTLA to neurodegenerative processes (Henon et al., 1998; Cordoliani-Mackowiak et al., 2003; Firbank et al., 2007), we investigated Alzheimer pathology in the hippocampal formation and entorhinal cortex of brain tissues obtained from the CogFAST (Cognitive Function after Stroke) Newcastle post-stroke cohort. Our objective was to quantify the presence of markers of amyloid pathology (total Aβ, Aβ-40, Aβ-42, and soluble Aβ) and a marker of hyperphosphorylated tau pathology in post-stroke subjects with and without dementia compared to aging controls, Alzheimer's disease (AD) and mixed AD and vascular dementia (AD-VaD). We hypothesized that in tandem with MTLA, hippocampal Alzheimer pathology would be differentially expressed in demented and non-demented stroke survivors in comparison with other dementias and aging controls.

## Methods

### Subjects

Ninety-four human post-mortem brains were retrieved from the Newcastle Brain Tissue Resource (NBTR) at the Campus for Ageing and Vitality, Newcastle University, UK. Table 1 provides details of the demographic, cognitive and pathologic characteristics of the subjects. Post-stroke subjects in the longitudinal CogFAST-Newcastle Study were classified based on the performance at the last cognitive assessment before death. They were classified as post-stroke non-demented (PSND) if CAMCOG score was >80 and Clinical Dementia Rating (CDR) was less than 1, but as post-stroke demented (PSD) if CAMCOG score was <80 and CDR score was >1 (Allan et al., 2011). Control subjects were historical subjects that had no significant evidence of cognitive impairment upon scrutiny of their medical records and whose post-mortem brain tissue was considered devoid of sufficient vascular or degenerative pathologies beyond the threshold for assigning a specific pathologic diagnosis (Kalaria et al., 2004; Gemmell et al., 2012).

**Table 1:** Characteristics of study subjects.

**Variable**	**Control**	**PSND**	**PSD**	**VaD**	**AD**	**AD_VaD**
Number of cases (*N*)	12	23	15	17	14	13
Age, years	79.1 ± 6.8	83.7 ± 3.9	87.3 ± 5.9	85.1 ± 6.4	83.5 ± 5.9	84.8 ± 5.7
Gender (M/F)	7/5	13/10	6/9	7/10	8/6	/7
MMSE score	N/A	27.1 ± 1.6	17.5 ± 3.7^*^	NCD	NCD	NCD
CAMCOG total score	N/A	89.1 ± 5.1	63.6 ± 13.5^*^	NCD	NCD	NCD
Braak Stage median (range)	2.0 (1–4)	2.0 (0–5)	3.0 (0–4)	2.0 (1–4)	5.5 (4–6)	5.0 (1–6)^*^
CERAD score Median (range)	NPD	2.0 (0–2)	1.0 (0–3)	1.0 (0–2)	3.0 (3–3)^*^	3.0 (1–3)^*^
Thal Stage-median (range)	NPD	3.0 (2–4)	1.0 (0–3)^*^	2.0 (0–3)	4.0 (3–4)^*^	4.0 (3–4)^*^
Vascular Score total-median (range)	NPD	13.0 (7–16)	12.0 (7–18)	14.0 (12–18)	NPD	12.0 (7–15)
Vascular Score-hippocampus Median (range)	N/A	2.0 (1–3)	2.0 (1–3)	N/A	N/A	N/A
ApoE Genotypes (%)	N/A	€3/3(43.7; €3/4, 2/4(50.0) Others (6.3)	€3/3 (53.8); €3/4 (23.1) €2/3 (16.7) Others (6.4)	N/A	N/A	N/A
Time from stroke to death (months)	N/A	60.7 ± 47.4	59 ± 21.8	N/A	N/A	N/A
Previous Stroke (% of cases)	N/A	Yes (52.6), No (42.1), Unknown (5. 3)	Yes (30.8), No (61.5), Unknown (7.7)	N/A	N/A	N/A
Location of lesion (% cases)	N/A	Parietotemporal (17.4), deep WM (13.0), cerebellum (8.7%) unknown (60.9)	Parietotemporal (35.7), deep WM (21.4), cerebellum (7.1), unknown (35.7)	N/A	N/A	N/A
Side of Lesion (% cases)	N/A	Left (8.7), Right (21.7), Both (26.1), None (26.1), Unknown (17.4)	Left (35.7), Right (21.4), Both (14.3), None (21.4), Unknown (7.1)	N/A	N/A	N/A
Vascular territory	N/A	MCA (30.4%), PCA (8.7), Unknown (60.9)	MCA (57.1), Vertebrobasilar (7.1), Unknown (35.7)	N/A	N/A	N/A

*Values show mean ± SD unless otherwise indicated. Post-mortem delay between autopsy and fixation of tissue (hours) and Fixation length (weeks) ranged 24–92 h and 6–34 weeks, respectively. There was no correlation between these variables and presence of pathology*.

* *p < 0.001. AD, Alzheimer's disease; AD_VaD, Mixed AD_VaD; PSND, MCA; Middle cerebral artery; N/A, Not Applicable NCD, No Cognitive Data; NPD, No Pathological Data PCA, Posterior cerebral artery; PMD, Postmortem delay; PSD, Post-stroke demented; Post-stroke non-demented; VaD, Vascular dementia*.

Autopsies were performed between 24 and 92 h after death and brains were fixed for between 6 and 34 weeks. Cognitive scores on the Mini-Mental State Examination (MMSE) and Cambridge Cognitive Examination (CAMCOG) proximate to death as well as APOE genotypes were retrieved from clinical and research records of the subjects in the CogFAST-Newcastle cohort. The CogFAST-Newcastle Study and ancillary studies had ethical approval from the local Newcastle Ethical committees and participants gave written consent for brain tissue donation. Use of brain tissue was also approved by the local Ethical committees (Newcastle upon Tyne Hospitals National Health Service Trust, UK) and the committee of the NBTR.

Post-mortem reports were retrieved for all the cases used in this study. Primary neuropathological diagnoses were made from brain tissue sampled at several coronal levels (Perry and Oakley, 1993) to check for pathological changes consistent with AD, VaD and mixed AD_VaD in accordance with established pathologic diagnostic criteria (Hyman and Trojanowski, 1997; Kalaria et al., 2004), and following macroscopic and microscopic post-mortem examination of the brain tissue. Haematoxylin and Eosin was utilized as a standard stain for a general neuropathologic structural evaluation of the brain, and for the detection of infarcts and rarefactions. Gallyas and Bielschowsky's silver impregnation stains and AT8 immunohistochemistry were used to evaluate “CERAD” neuritic plaques and neurofibrillary tangles according to the methods of Braak (Braak and Braak, 1991; Mirra et al., 1991). In addition, vascular lesions (cortical and sub-cortical infarcts, border-zone infarcts, strategic infarcts, lacunar infarcts (<15 mm), microinfarcts (<5 mm) and mild, moderate and severe cerebral amyloid angiopathy were recorded (Kalaria et al., 2004). Final diagnoses were assigned during monthly clinicopathologic consensus meetings. A final diagnosis of VaD was made if there was clinical evidence of dementia (DSM IV) and pathologic evidence of multiple or cystic infarcts, lacunes, micro-infarcts, small vessel disease in the absence of severe degenerative pathology (Braak Stage <III) (Kalaria et al., 2004). Subjects were assigned mixed AD_VaD if there was pathologic evidence of cerebrovascular disease in the presence of significant AD pathology (Braak Stage V or VI) and moderate to severe CERAD scores. A diagnosis of AD was assigned when there was significant Alzheimer pathology-Braak V–VI, moderate to severe CERAD score and absence of significant vascular pathology.

### APOE genotyping

APOE genotyping was undertaken in the CogFAST cohort only (PSND and PSD) as shown in Table 1 using restriction enzyme isoform genotyping as previously described (Hixson and Vernier, 1990; Rowan et al., 2005). In brief, ApoE restriction isotyping used oligonucleotides to amplify apolipoprotein E gene sequences containing amino acid positions 112 and 158. The amplification products were digested with HhaI and subjected to electrophoresis on polyacrylamide gels. Each of the isoforms was distinguished by a unique combination of HhaI fragment sizes that enabled unambiguous typing of all homozygotic and heterozygotic combinations. HhaI cleaves at GCGC encoding 112arg (E4) and 158arg (E3, E4), but does not cut at GTGC encoding 112cys (E2, E3) and 158cys (E2).

### Immunohistochemistry

Paraffin embedded brain tissue blocks taken from relevant coronal levels of the Newcastle Brain Map (Perry and Oakley, 1993) and containing the hippocampal formation and entorhinal cortex were cut into 10 μm serial sections using a rotary microtome. Sections were mounted on slides coated with 2% APES (3-aminopropyltrethoxysilane) solution in acetone, and dried in a pre-heated oven at 600C for 30 min. The cut sections were then serially immunostained in duplicates with primary antibodies to different amyloid-β species and tau (Table 2). The sections were first de-paraffinized in two sequential solutions of Xylene for 15 min and then rehydrated using decreasing concentrations of ethanol (100, 95, 70, and 50%) to deionized water. Antigen retrieval was performed for Aß-immunolabeling using formic acid-pretreatment for 4 h and for tau immunolabeling using heat in the form of microwaving sections for 10 min in a solution of 0.01M citrate buffer (PH 6.0). The buffer was brought to boil in the microwave, slides were added, buffer was microwaved at 450W for 10 min, and the solution was then allowed to cool for 20 min following which slides were transferred to deionized water. Non-specific reaction was quenched with 0.9% hydrogen peroxide (unless otherwise stated.) in 5 Mm Tris buffered Saline (TBS) solution (pH 7.6) for 15 min in order to remove endogenous peroxidise. Non-specific antigens were blocked using normal horse serum (anti-mouse antibody: 4G8, NU-1 and AT8) and normal goat serum (anti-rabbit antibody: T-40 and T-42 for 60 min. The slides were then incubated for 2 h at room temperature (AT8) or overnight at 40C (4G8, T-40, T-42, and NU-1) with the primary antibody diluted to specific concentration with buffer: total amyloid β (4G8, 1: 1000, Mouse monoclonal, Signet 9220-10), amyloid β-42 (T-42, 1: 5000, Rabbit polyclonal, gift; H. Mori, Japan), amyloid β-40 (T-40, 1: 5000, Rabbit polyclonal, gift; H. Mori, Japan), soluble amyloid oligomer (NU-1, Mouse monoclonal, gift: C. Klein, US) and antibody to hyperphosphorylated tau (AT8, 1: 1000, Mouse monoclonal Autogen Bioclear). After washes in buffer, biotinylated secondary antibody was applied to the sections with the blocking serum for 30 min, followed by the addition of the streptavidin/biotinyl-horseradish peroxidase (HRP) complex (SABC) for 30 min to remove excess secondary antibody. Finally, the slides were immersed in a 0.025% diaminobenzidine (DAB) solution for a variable short period of time to visualize the positive antibody reaction. Sections were then rinsed in water and counterstained in haematoxylin as indicated. Sections were then dehydrated back through graded alcohols, cleared in xylene and mounted with glass coverslips using DPX mounting medium (Sigma, UK). After each step, with the exception of the blocking stage, sections were rinsed in buffer (TBS) three times for 5 min each. All immuno-histochemical protocols included a positive control and a negative control for which the primary antibody is omitted. All the antibodies used in the analysis have been previously well characterized. We have published on use of these antibodies as described in our previous publications (Chang et al., 2003; Lewis et al., 2006; Ndung'u et al., 2012; Mukaetova-Ladinska et al., 2015).

**Table 2:** Summary of immunoperoxidase staining with DAB as chromogen.

**Antibody**	**Nature /Source**	**Antigen retrieval**	**Assay buffer**	**Block**	**Dilution**	**Secondary Antibody**	**SABC**
4G8	Monoclonal/Mouse	Concentrated formic acid	TBS	1.5% NoHoS × 1 h	1:1000 O/N	Anti-mouse	Yes
T-42	Polyclonal/Rabbit	Concentrated formic acid	TBS	1.5% NoGoS × 1 h	1:5000 O/N	Anti-rabbit	Yes
T-40	Polyclonal/Rabbit	Concentrated formic acid	TBS	1.5% NoGoS × 1 h	1:5000 O/N	Anti-rabbit	Yes
NU-1	Monoclonal/Mouse	Concentrated formic acid	TBS	–	1:3200 O/N	Anti-mouse	Yes
AT8	Monoclonal/Mouse	Microwave/Citrate buffer	TBS	1.5% NoHoS × 1 h	1: 1000 2 h Room Temperature	Anti-mouse	Yes

*NoHoS, Normal horse serum; NoGoS, Normal goat serum; O/N, Overnight; SABC, avidin biotin complex; DAB, diaminobenzidine*.

### Microscopy and image analysis

Stained sections were examined and digitized using a Zeiss Axioplan 2 research grade microscope coupled to an Infinity 2 camera. Magnification was set at X10 for the hippocampal sub-regions CA1, CA2 and CA3, and X5 for the subiculum and entorhinal cortex (EC). Five images were taken at random from the CA1, CA3 and subiculum, 3 images from CA2 and 4 × 3 from the EC from the pial surface to the white matter. Approximately 2,820 images were taken.

Images were analyzed using Image Pro Plus 6.3 (Media Cybernetics, Silver Spring, MD, USA). The area of interest (AOI) was delineated on-screen using the wand tool and the size of the area selected was recorded. Histogram based analysis was used to measure the number of pixels stained (“per area,” PA) and intensity of stain (integrated optical density, IOD), as determined by the operator. Background was excluded by highlighting only the positive staining. To standardize the measurements, the range of stain intensity for each AOI was determined between 0 and 255 (where 0 = black and 255 = white, as set up for the Image Pro Plus Histogram based analysis). Using the sum of each of the three measures, further calculations were undertaken to generate the metrics of immunostaining: Percent of the area of interest positively stained, “Percent area (%PA)” = Per Area × 100; Mean intensity of stain per pixel, “Integrated Optical Density (IOD)” = 255 – (sum IOD/area). The mean % PA and IOD were then calculated for each subject by computing averages from the images taken from each hippocampal sub-region.

### Statistics

Statistical analysis was carried out using the IBM SPSS software (version 19.0). The Kolmogorov-Smirnov Test was used to establish normality of data. Comparisons across groups of cases and across sub-regions were performed using parametric tests (ANOVA for group means and Tukey *post-hoc* analysis for between-group differences) and non-parametric tests Kruskal–Wallis and Mann–Whitney *U*-tests) for non-normally distributed dataset. The relationship among markers, and with demographic, cognitive and pathological variables were assessed using Pearson's correlation coefficient (*r*) or Spearman's correlation (rho) as necessary depending on the normality of the dataset. Appropriate power calculation was performed using the G^*^Power software (Faul et al., 2007) at significance level, α-level = 0.05 and assuming a moderate effect size Cohen's d = 0.4.

## Results

### Characteristics of study subjects

The demographic, cognitive and pathological characteristics of the study participants [non-demented (PSND), demented (PSD) subjects from the CogFAST-Newcastle study; Control, VaD, AD and mixed AD-VaD groups] are shown in Table 1. There were no significant differences in the age (*p* = 0.786), gender distribution (*p* = 0.493), post-mortem delay (*p* = 0.902) and length of fixation of tissues (*p* = 0.589) across the groups. However, the PSND group had significantly higher scores on the cognitive batteries MMSE and CAMCOG compared to the PSD group (*p* < 0.05). Similarly, Braak stage, CERAD score and Thal stage were significantly higher in the AD, AD-VaD groups compared to the VaD and post-stroke groups (PSND and PSD) (*p* < 0.05). Hippocampal vascular scores were similar among the PSND, PSD, VaD and AD-VaD groups. The distribution of the ε3 and ε4 alleles of *APOE* were not significantly different between the PSND and PSD groups (Fisher's exact test = 2.13; *p* = 0.249). Given a total sample size of 94, a significance level, α = 0.05 and assuming a moderate effect size Cohen's *d* = 0.4, 6 sub-groups and 5 degrees of freedom, the computed Power (1-β) = 0.8424 using the G^*^Power software (Faul et al., 2007).

### Quantification of amyloid (Aβ) burden

**Figure 1:**
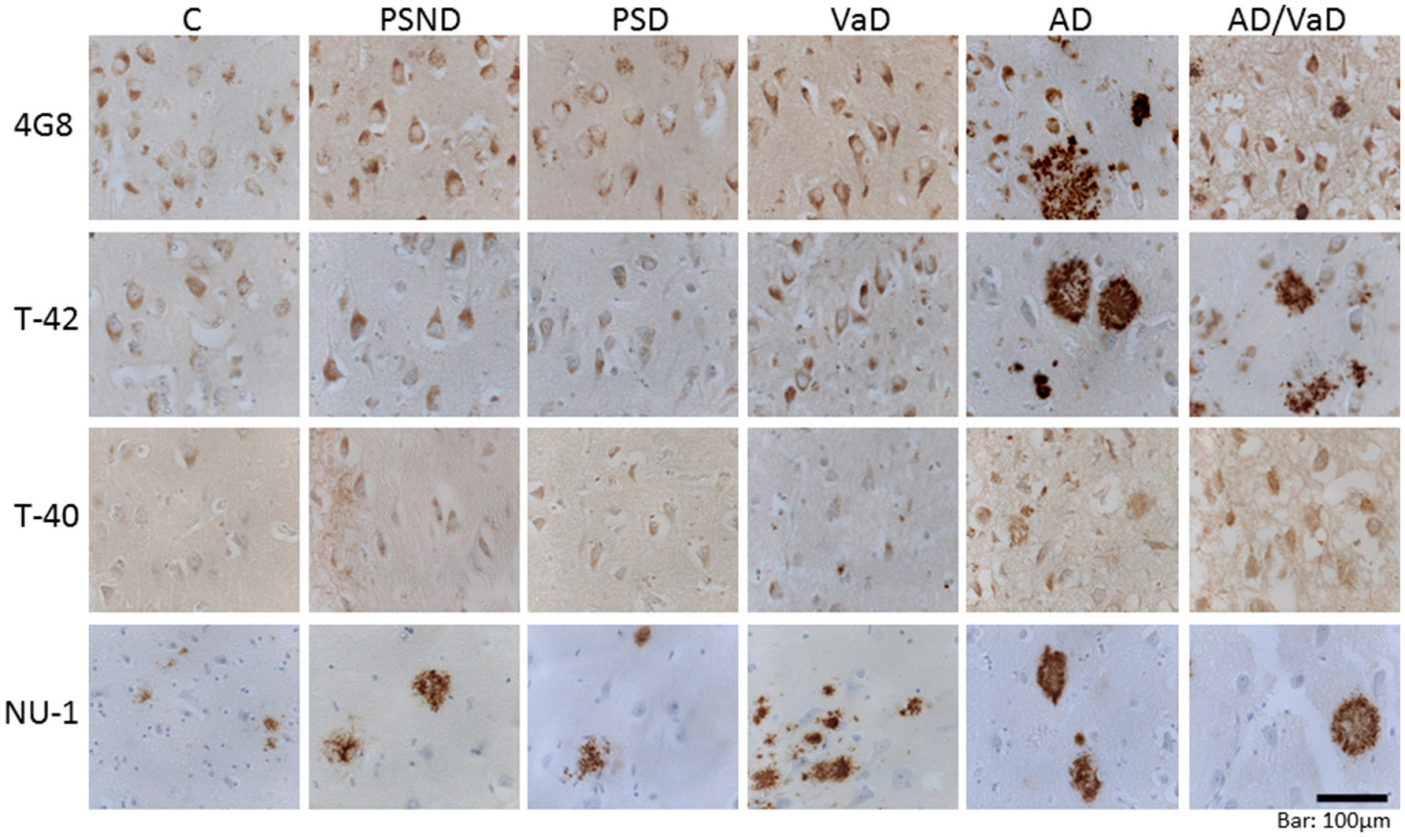
Illustrative images of amyloid pathology in the CA1 sub-region across different markers and across disease groups and aging controls. There is higher expression of amyloid in the AD and AD_VaD groups compared with the VaD, PSD, and PSND and control groups. The level of amyloid immunoreactivity is similar between 4G8 and T-42 but much lower in T-40 and NU-1.

In quantifying amyloid burden both parenchymal as well as vascular amyloid immunoreactivities were incorporated in order to capture the total quantity of the different species of amyloid detected within the defined area of interest as previously performed (Lewis et al., 2006). Figure 1 illustrates the immunostaining pattern with antibodies to various Aβ species in serial sections across the CA1 sub-region of the hippocampus.

#### Immunolabeling of total Aβ with 4G8 antibody

Distribution of 4G8 antibody immunostaining dataset assessed by Kolmogrov-Smirnov test showed non-normal distribution. Spearman's rank correlation analysis revealed no significant associations between post-mortem delay, length of fixation period and 4G8 immunoreactivity (IR) measures in the hippocampal sub-regions and entorhinal cortex. Furthermore, there was significant positive correlation between scores on the semi-quantitative amyloid rating scales of CERAD, Thal, Braak and tau stages with the metrics of 4G8 total IR in the entorhinal cortex, subiculum and CA1 sub-regions (Table 3) suggesting good agreement between semi-quantitative and quantitative measures of amyloid and tau quantification.

**Table 3:** Correlation Matrix of sub-regional total Aβ immunoreactivity, CERAD score, Thal stage, Braak stage and tau stage.

**Spearmans rho**	**CA1α**	**CA2α**	**CA3α**	**SBα**	**ECα**	**Braakβ**	**Tauβ**	**Thalβ**	**CERADβ**	
CA1α	ρ	1.000								
	p									
CA2α	ρ	0.403^**^	1.000							
	p	0.000								
CA3α	ρ	0.444^**^	0.665^**^	1.000						
	p	0.000	0.000							
SBα	ρ	0.618^**^	0.169	0.243^*^	1.000					
	p	0.000	0.174	0.048						
ECα	ρ	0.490^**^	0.094	0.220^*^	0.758^**^	1.000				
	p	0.000	0.432	0.061	0.000					
Braakβ	ρ	0.420^**^	0.009	0.166	0.404^**^	0.429^**^	1.000			
	p	0.001	0.946	0.222	0.003	0.001				
Tauβ	ρ	0.316^**^	0.160	0.019	0.334^**^	0.474^**^	0.582^**^	1.000		
	p	0.008	0.194	0.878	0.008	0.000	0.000			
Thalβ	ρ	0.442^**^	0.223^†^	0.001	0.633^**^	0.653^**^	0.680^**^	0.685^**^	1.000	
	p	0.000	0.059	0.995	0.000	0.000	0.000	0.000		
CERADβ	ρ	0.341^*^	0.045	0.095	0.492^**^	0.524^**^	0.721^**^	0.597^**^	0.706^**^	1.000
	p	0.11	0.749	0.492	0.000	0.000	0.000	0.000	0.000	

α designates total Aβ (4G8) immunoreactivity in the CA1, CA2, CA3, subiculum (SB) and entorhinal cortex (EC) sub-regions of the hippocampus while β depicts CERAD score, Thal stage, Braak stage and tau stage. Statistical significance designated by the following p-values:

** p < 0.01;

* p < 0.05;

† *p < 0.1. Abbreviations: CERAD, Consortium to Establish a Registry for Alzheimer's Disease*.

**Figure 2:**
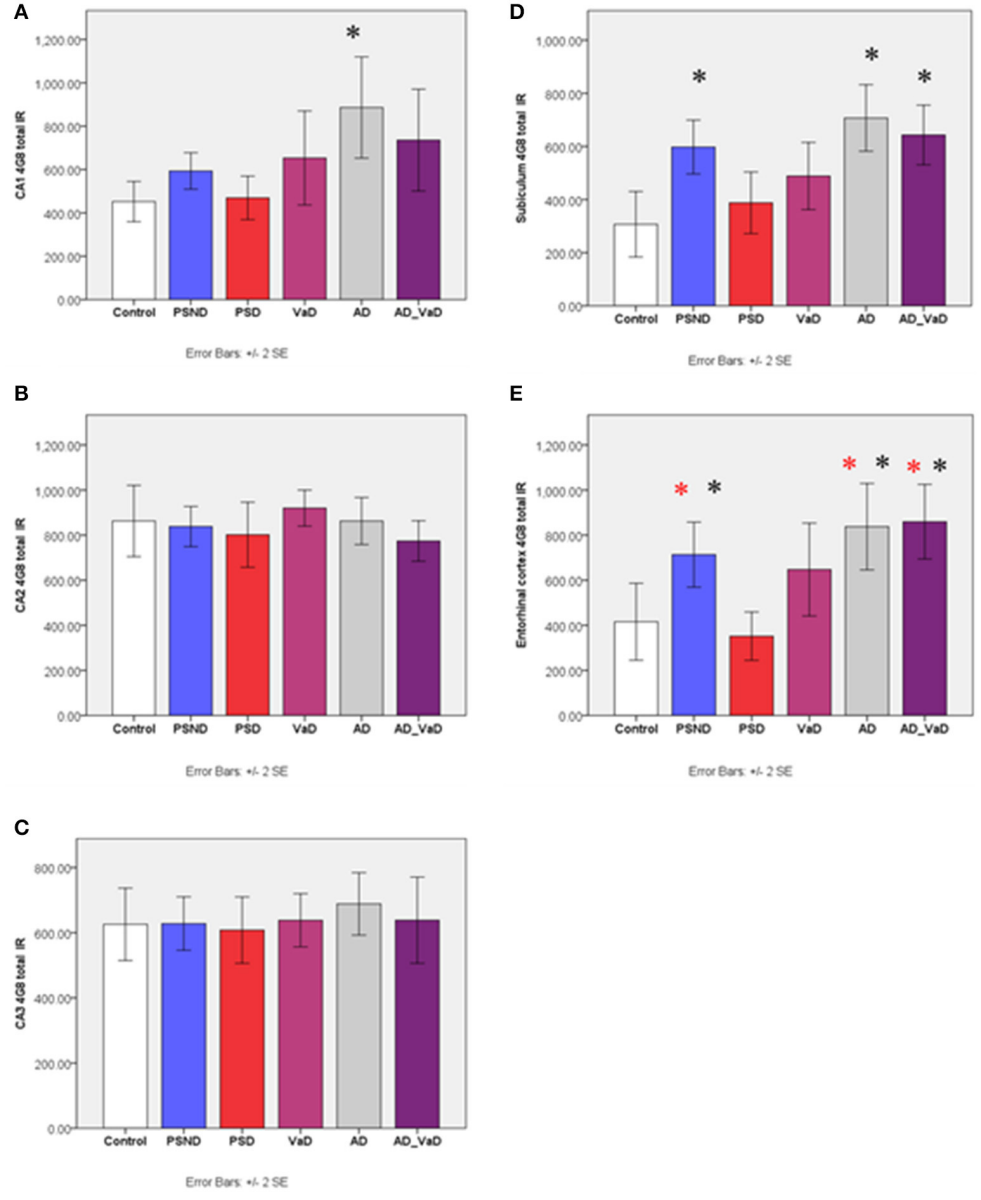
Bar graphs showing the distribution of total Aβ IR across hippocampal sub-regions **(A)** CA1, **(B)** CA2, **(C)** CA3, **(D)** Subiculum, **(E)** Entorhinal cortex in Controls, PSND, PSD, VaD, AD and AD_VaD. Bars show ± 2 SEM ^*^Mann Whitney *U*-test was used to compare means of each group. ^*^p < 0.05; + *p* < 0.1 (in comparison with the control group). *(red) showed significant difference from PSD group. IR (immunoreactivity).

In the CA1 region, total Aβ IR varied significantly across groups (*p* = 0.008, Kruskal-Wallis Test) (Figure 2). Between group differences assessed with Mann–Whitney *U*-Test showed that compared to the control group, total AβIR was significantly higher in AD (*p* = 0.002), showed a trend with AD-VaD (*p* = 0.064) and PSND (*p* = 0.076) but not significantly different between PSD and VaD. In the CA2 and CA3 regions, there was no significant variation across disease groups. In the subiculum, there was significant variation across disease groups and controls (*p* < 0.001, Kruskal–Wallis Test) with total IR being significantly higher in the AD (*p* = 0.001), AD-VaD (*p* = 0.008) and PSND (*p* = 0.015) groups but not significantly different between PSD and VaD. In the entorhinal cortex, total Aβ IR similarly showed significant variation across the sub-region (*p* < 0.001, Kruskal–Wallis Test) with values significantly higher in AD (*p* = 0.022), AD-VaD (*p* = 0.016) than in controls while PSD was significantly lower than PSND (*p* = 0.019), AD-VaD (*p* = 0.002) and AD (*p* = 0.002) (Figure 2).

#### Aβ-42 immunohistochemistry with T-42 antibody

**Figure 3:**
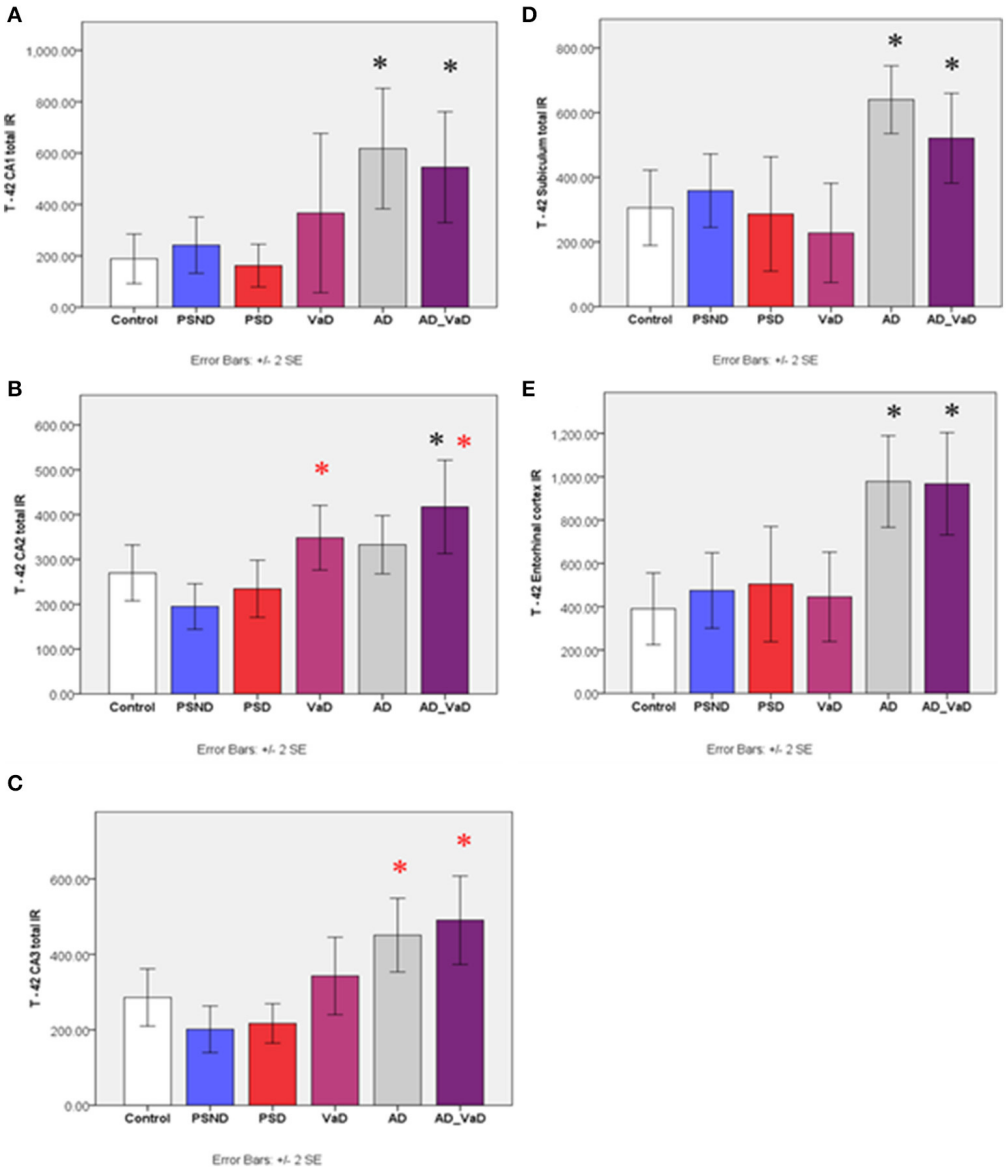
Bar graphs showing the distribution of Aβ-42 IR across hippocampal sub-regions **(A)** CA1, **(B)** CA2, **(C)** CA34, **(D)** Subiculum, **(E)** Entorhinal cortex in Controls, PSND, PSD, AD, VaD, and AD_VaD. Bars show ± 2 SEM ^*^Mann Whitney *U*-test was used to compare means of each group. ^*^p < 0.05; + *p* < 0.1 (in comparison with control group). *(red) showed significant difference from PSND group. IR (immunoreactivity).

**Figure 4:**
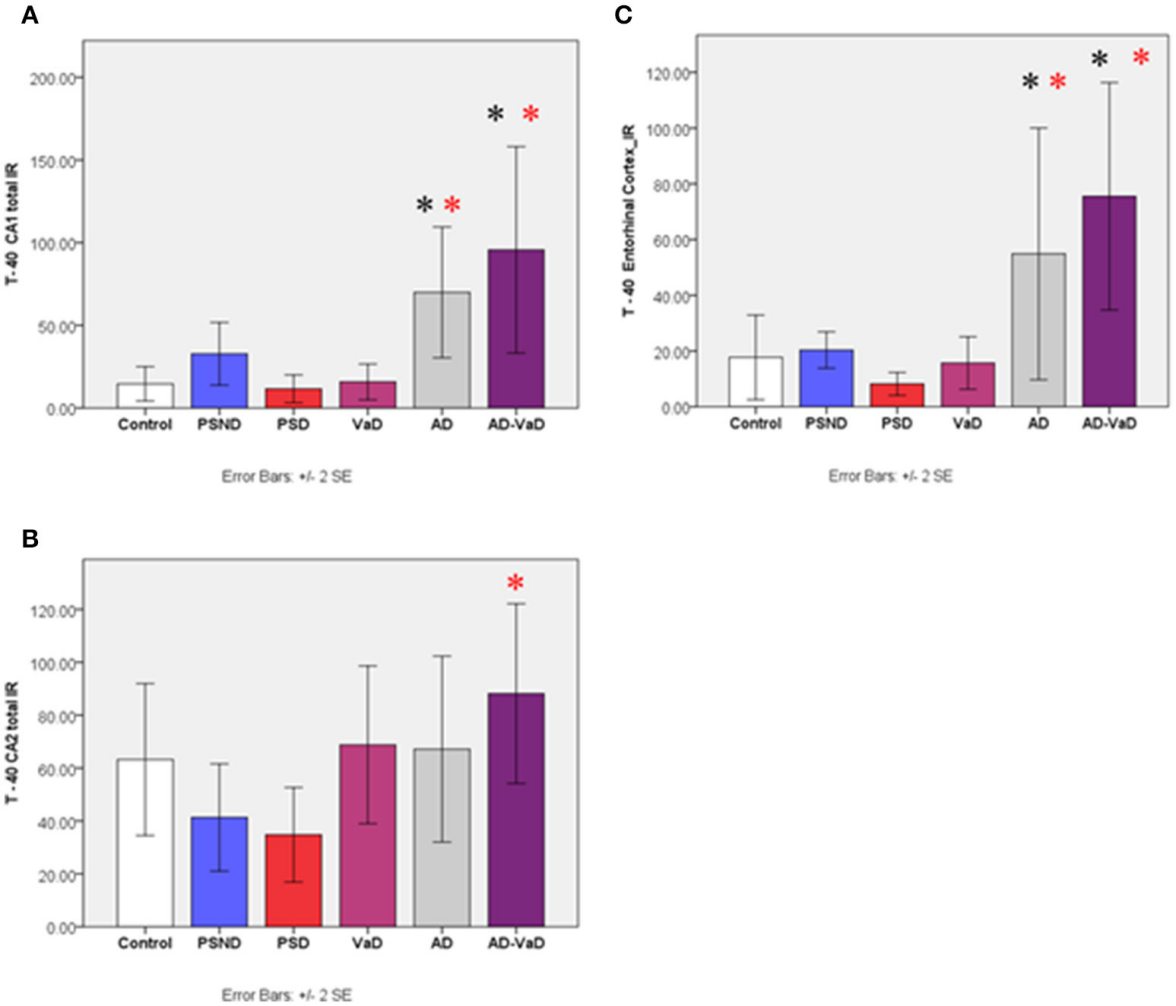
Bar graphs showing the distribution of Aβ-40 IR across hippocampal sub-regions **(A)** CA1, **(B)** CA2, **(C)** Entorbinal cortex in Controls, PSND, PSD, AD, VaD and AD_VaD. Bars show ± 2 SEM ^*^Mann Whitney *U*–test was used to compare means of each group. ^*^p < 0.05; + *p* < 0.1 (in comparison with the control group). *(red) showed significant difference from PSD group. lR (immunoreactivity).

**Figure 5:**
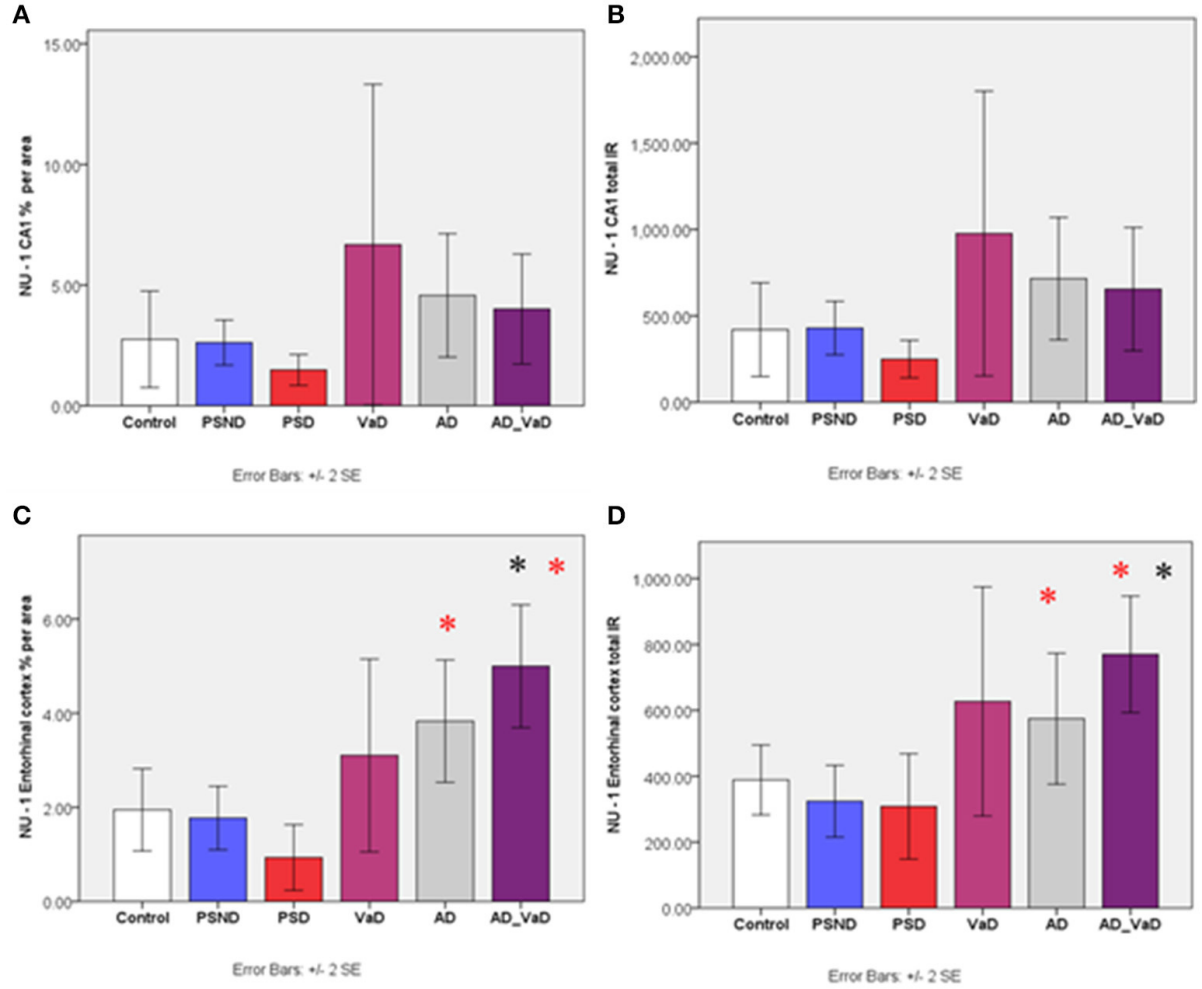
Bar graph shows percentage area and immunoreactivity of soluble Aβ (IR) across the CA1 region **(A,B)** and Entorhinal cortex **(C,D)** respectively in Control PSND, PSD, VaD, AD, and AD_VaD. Bars show ± 2 SEM ^*^Mann Whitney *U*-test was used to compare means of each group. ^*^p < 0.05 (in comparison with the ^*^control group and *PSD group). There were no significant differences across groups in the CA1, CA2, and CA3 regions (*p* > 0.05, Kruskal–Wallis Test).

In the CA1 region, *A*β*-42* immunoreactivity varied significantly across groups (*p* < 0.001, Kruskal-Wallis Test) (Figure 3). Between group differences assessed with Mann–Whitney *U*-Test showed that compared to the control group, T-42 immunoreactivity was significantly higher in AD (*p* = 0.002) and AD-VaD (*p* = 0.005), but not significantly different from VaD, PSND and PSD (Figure 3). In the CA2 region, immunoreactivity varied across disease groups (*p* = 0.001, Kruskal–Wallis Test) with the IR being significantly higher in AD-VaD (*p* = 0.046) and VaD (*p* = 0.014) groups compared to the control and PSD groups respectively (Figures 4B, 5). In the CA3 region, values revealed normal distribution by Kolmogorov–Smirnov test of normality and ANOVA showed significant variation of *A*β*-42* immunoreactivity across the regions (*p* < 0.001). Compared to the PSND group, immunoreactivity was significantly higher in AD (*p* < 0.001) and AD-VaD (*p* = 0.002) groups but not significantly different from control PSD and VaD groups (Figure 3). In the subiculum, *A*β*-42* immunoreactivity varied significantly across groups (*p* < 0.001) and was significantly higher in the AD (*p* = 0.001) and AD-VaD (*p* = 0.008) groups compared to the control and VaD groups respectively. However, the VaD groups did not differ significantly from the PSND and PSD groups respectively (Figures 4, 5). Similarly, *A*β*-42* immunoreactivity varied significantly across the entorhinal cortex (*p* < 0.001) and was significantly higher in the AD (*p* = 0.001) and AD-VaD (*p* = 0.001) groups but not different from the VaD, PSND and PSD groups.

#### Aβ-40 immunoreactivity with T-40 antibody

Immunostaining and distribution of T-40 antibody across disease groups varied significantly across the sub-regions (*p* = 0.001, Kruskal–Wallis Test). In CA1, the IR in the control group was not significantly different from that of PSND, PSD and, VaD but was significantly lower than AD (*p* = 0.004) and AD-VaD (*p* = 0.010). There was no difference between the PSND and PSD groups. In CA2, the control group IR was lower than AD, VaD and AD-VaD although this did not attain statistical significance (*p* > 0.05). However, VaD (*p* = 0.005) and AD-VaD (*p* = 0.006) groups were significantly higher than the PSD group. The PSND group did not differ from the PSD group (Figure 4). In the entorhinal cortex, *A*β*-40* IR was significantly higher in the AD and AD-VaD groups compared to the control and PSD groups (*p* < 0.05, Kruska–Wallis Test). There was no significant variation in the CA3 region and subiculum.

We further explored the relationship of Aβ-42 and Aβ-40 based on the relative immuno-reactivities of T-42 and T-40 in the CA1 sub-region. The ratio varied between 1.17 and 748. 39 and the mean value was least in the control group and progressively increased in the PSND, PSD, VaD and AD groups to attain a maximum value in the AD-VaD group (Data not shown).

#### Soluble Aβ-immunoreactivity with NU-1 antibody

There were no significant differences in the percentage area and total immunoreactivity of NU-1 across the disease groups in the CA1, CA2, and CA3 regions (*p* > 0.05, Kruskal Wallis Test). However, in the subiculum and entorhinal cortex, the immunoreactivity varied significantly (*p* < 0.05 Kruskal–Wallis Test). Between group analysis showed significantly higher immunoreactivity in the AD-VaD (*p* = 0.007 Mann–Whitney U) group compared to the control group, and in the AD-VaD (*p* = 0.008) and AD (*p* = 0.040) compared to the PSD group (Figure 5). The PSND and PSD groups showed no significant differences.

#### Hyperphosphorylated tau immunoreactivity with AT8 antibody

**Figure 6:**
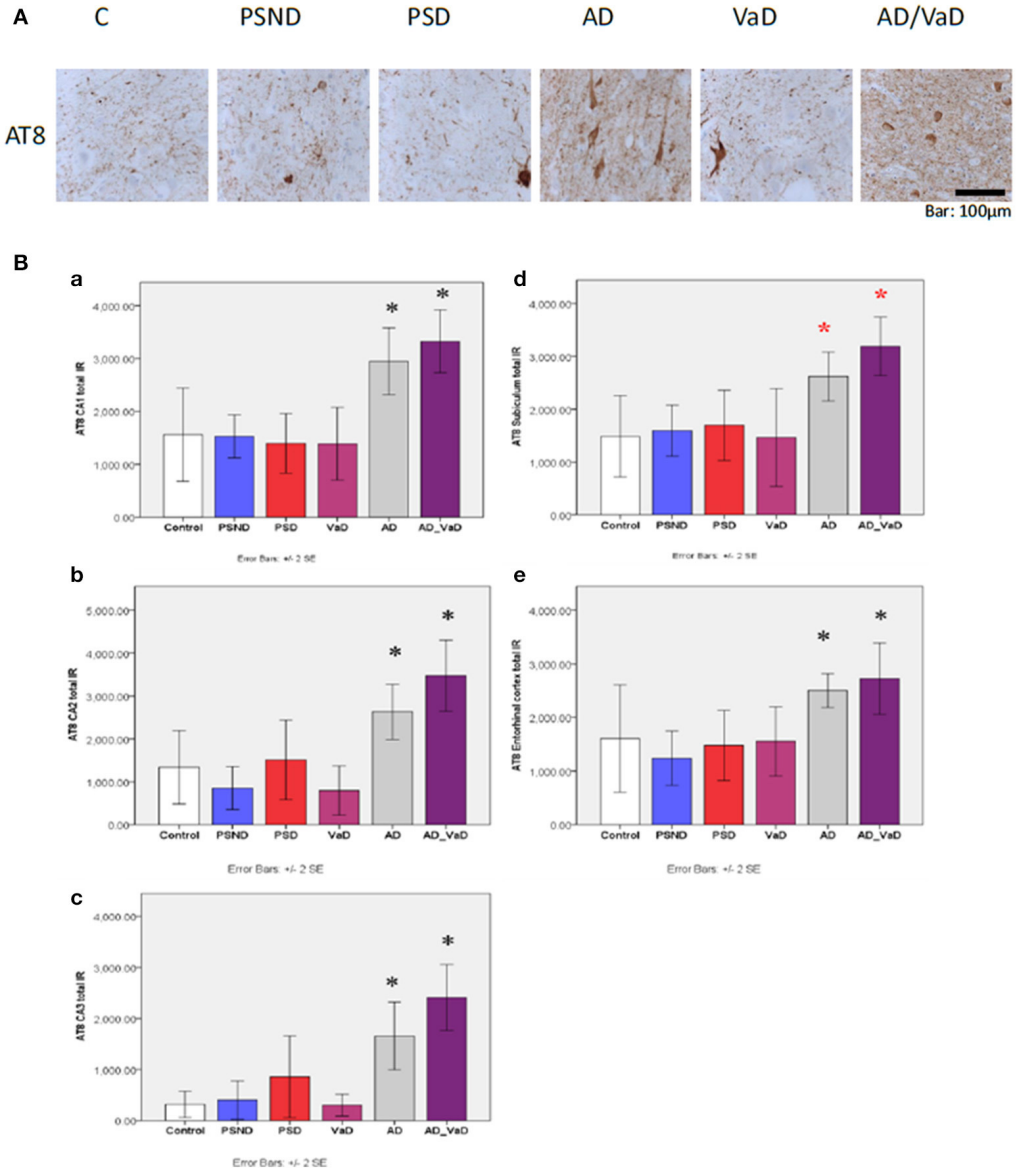
**(A)** Hyperphosphorylated tau (AT8) immunoreactivities showing neuropil threads, pretangles and tangles in hippocampal sub-region CAl. AT8 immunoreactivity is relatively higher in AD and AD_VaD compared to other groups. **(B**) Bar graph showing the distribution of AT8 IR across hippocampal sub-region. **(a)** CA1, **(b)** CA2, **(c)** CA3, **(d)** Subiculum **(e)** Entorhinal cortex in Controls. PSND. PSD. AD. VaD and AD_ VaD. Bars show ± 2 SEM ^*^Mann Whitney *U*-test was used to compare means of each group. ^*^p < 0.05; (in comparison with the control group). *(red) showed significant difference from PSND group.

Figure 6A shows tau (AT8) immunoreactivity across disease groups in the hippocampal subregion CA1 while Figure 6B shows the quantification across different subregions. Across disease groups, AT8 immunoreactivity was highest in AD and AD_ VaD groups in comparison to each of the other groups of PSND, PSD, VaD and Controls (*p* < 0.05). There was slightly higher AT8 immunoreactivity in PSD compared to the PSND group in the CA2 and CA3 sub-regions (Figure 6B) although the difference did not attain statistical significance.

#### Influence of APOE ε4 genotype on amyloid and tau accumulation in post-stroke sub-cohort

**Figure 7:**
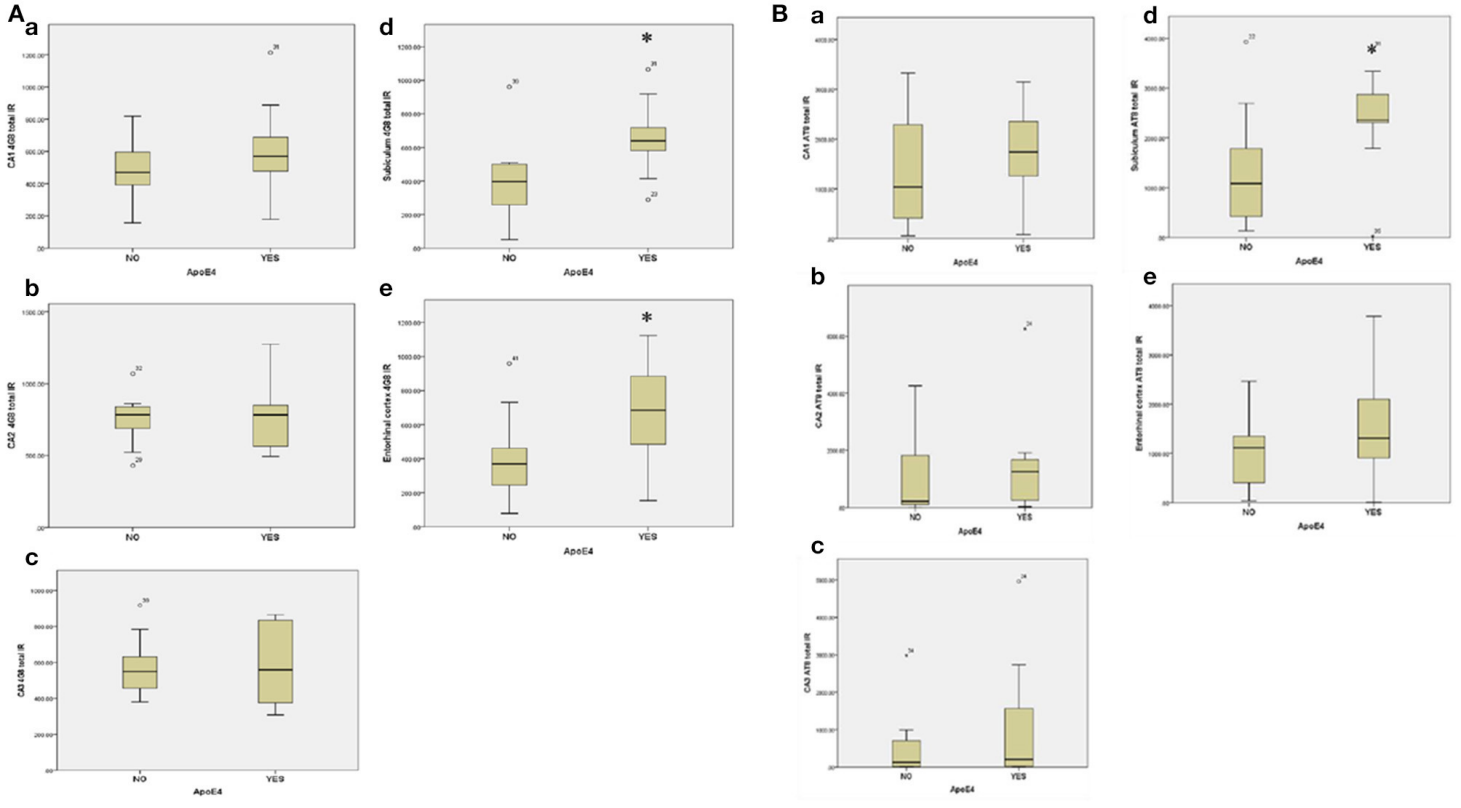
**(A)** Box Plots showing the influence of Apo E ε4 on total amyloid deposition across hippocampal sub-regions **(a)** CA1, **(b)** CA2, **(c)** CA3, **(d)** Subicuhun, and **[e]** Emorlrinal conex in the post-stroke sub-cohort Mann–Whitney *U*-test was used to compare the mean 4G8 total immunoreactivity between the Apo E ε4 positive and negative groups respectively. ^*^p < 0.05. **(B**) Box Plot showing the influence of Apo E ε4 on total tau (AT8) deposition across hippocampal sub-regions **(a)** CA1 **(b)** CA2 **(c)** CA3 **(d)** Subiculum and **(e)** Entorhinol cortex in the post-stroke sub-cohort. Mann Whitney *U*-test was used to compare mean AT8 total immunoreactivity between the Apo E ε4 positive and negative groups respectively. ^*^p < 0.05.

Eight out of 16 subjects with PSND (50%) were APO ε4 positive compared to 3 out of 13 PSD cases (23.1%). Overall, 11 out of 29 (37.9%) post-stroke subjects were *APOE* ε4 positive. Evaluation of the influence of *APOE* ε4 status on amyloid and tau deposition in the post-stroke sub-cohort revealed a statistically significant higher amyloid load in *APOE* ε*4* positive post-stroke subjects compared to *APOE* ε*4* negative subjects in the subiculum and entorhinal cortex respectively (*p* = 0.01) (Figure 7A). Presence of *APOE* ε*4* also influenced tau deposition (AT8 immunoreactivity) in the post-stroke cohort, the immunoreactivity being significantly higher in *APOE* ε*4* positive subjects in the subiculum (Mann–Whitney *U*-Test, *p* = 0.042) (Figure 7B).

#### Clinico-pathological correlations

We explored relationships between the various markers of Alzheimer pathology used in this study and cognitive scores of the post-stroke group utilizing Spearman correlation analysis. Measures of general cognitive functioning (MMSE and CAMCOG total) and functioning in the memory domain (CAMCOG memory) were correlated with two measures of Alzheimer pathological burden: Aβ-42 immunoreactivity (being the predominant β-amyloid species deposited in the hippocampus) and tau immunoreactivity measures in the CA1, subiculum and entorhinal cortex: sub-regions which demonstrated the most consistent patterns of variation of immunoreactivity across the hippocampal formation. Table 4 demonstrates significant correlation of only AT8 immunoreactivity in the subiculum with CAMCOG memory (rho = −0.425, *p* = 0.024), whereas there was no significant correlation with Aβ-42 immunoreactivity.

**Table 4:** Correlation matrix showing association of neuropathologic measures of amyloid and tau pathology with cognitive scores in selected hippocampal sub-regions and entorhinal cortex.

**Spearman's rho**		**CAMCOG memory**	**CAMCOG total**	**MMSE**	**CA1α**	**SBα**	**ECα**	**CA1β**	**SBβ**	**ECβ**
CAM_memory	ρ	1.000								
	p	.								
CAMCOG score	ρ	0.814^**^	1.000							
	p	0.000	.							
MMSE	ρ	0.790^**^	0.951^**^	1.000						
	p	0.000	0.000	.						
CA1α	ρ	−0.077	0.029	0.053	1.000					
	p	0.657	0.857	0.754	.					
SBα	ρ	−0.419^*^	−0.189	−0.047	0.793^**^	1.000				
	p	0.024	0.301	0.807	0.000	.				
ECα		−0.308	−0.064	−0.031	0.785^**^	0.686^**^	1.000			
	p	0.091	0.720	0.869	0.000	0.000	.			
CA1β	ρ	−0.046	0.109	0.054	0.656^**^	0.493^**^	0.567^**^	1.000		
	p	0.792	0.513	0.759	0.000	0.000	.000	.		
SBβ	ρ	−0.080	0.120	0.090	0.634^**^	0.563^**^	0.557^**^	0.779^**^	1.000	
	p	0.646	0.471	0.609	0.000	0.000	0.000	0.000	.	
ECβ	ρ	−0.033	0.087	0.082	0.566^**^	0.457^**^	0.557^**^	0.566^**^	0.671^**^	1.000
	p	0.849	0.606	0.641	0.000	0.000	0.000	0.000	0.000	.

## Discussion

We hypothesized that Alzheimer pathology would be differentially expressed in demented and non-demented post-stroke subjects in comparison to normal aging controls and other dementias. We found (1) amyloid beta deposition was not remarkably different between PSD and PSND groups despite the finding that MTLA was a significant feature in PSD subjects. (2) As expected, concentrations of total amyloid beta and amyloid β-42 were significantly greater in AD and mixed subjects but low in post-stroke subjects consistent with insufficient concentrations for a diagnosis of AD (3) an association between *APOE* ε*4* allele positivity and higher load of amyloid and tau pathology in the subiculum and entorhinal cortex of post-stroke cases (4) hyperphosphorylated tau immunoreactivity did not differ significantly between PSND and PSD groups and (5) poor correlation of cognitive measures with the burden of amyloid and tau pathology in post-stroke subjects.

### Amyloid accumulation, aging and cerebrovascular disease

Consistently across all the markers of amyloid pathology, we found evidence of increasing accumulation of amyloid in controls, post-stroke groups, AD and AD_VaD in that order. Our finding of amyloid accumulation in normal aging controls concurs with the biological phenomenon of aging-associated accumulation of amyloid that has been reported across species: in drosophilia (Rogers et al., 2012), mice (Yamada et al., 2011), non-human primates (Ndung'u et al., 2012) and man (Tomlinson et al., 1968; Katzman et al., 1988; Bennett et al., 2006; Lewis et al., 2006; Boyle et al., 2013b). This occurs as a result of aging-related compromise of the neurovascular unit resulting in increased production of amyloid and reduced clearance through the perivascular space (Iadecola, 2004; Kalaria, 2009; Kalaria et al., 2012). Amyloid accumulation in the post-stroke groups mirrored that in the VaD group and was less than in the AD and AD-VaD groups (Figures 2, 3) in consonance with the findings of Lewis et al. (2006) showing enhancement of amyloid accumulation in VaD possibly triggered by cerebral hypoxia consequent to cerebral vascular disease (Lewis et al., 2006; Kalaria et al., 2016). This is in tandem with previous findings of enhanced accumulation of amyloid in animal models of chronic cerebral hypoperfusion (Kalaria et al., 1993a; Yamada et al., 2011) as well as increased Pittsburgh Compound B (PIB) uptake in PSD subjects in a pilot study (Mok et al., 2010). In addition, this suggests that beyond age-associated accumulation of amyloid, cerebral vascular disorders including stroke do exacerbate brain amyloid deposition as previously demonstrated in brain tissue from hypertensive (Petrovitch et al., 2000) and diabetic subjects (Luchsinger, 2010). Although Marchant et al. failed to establish a direct relationship between CVD and Aβ using PIB-PET approach in a cohort of non-demented elderly subjects (Marchant et al., 2012), and further suggested that the PIB-PET amyloid measures did not influence cognition, the authors admitted that the limited statistical power of the study may have failed to detect any existent interaction. Utilizing a semi-quantitative approach, a neuropathological study of 484 post-mortem brains did not find a relationship between amyloid deposition and cerebrovascular lesions (Aho et al., 2006). The previous study by Lewis et al. (2006) and this current work have utilized sensitive quantitative approaches to detect amyloid load. Besides, findings from studies in non-demented elderly subjects may not necessarily simulate those in demented subjects with significant CVD as the mechanisms that produce cognitive decline and dementia may differ in different clinicopathological scenarios (Kalaria, 2012a,b).

Although the main Aβ variants detected in the human brain are Aβ1–40 and Aβ1–42, a significant proportion consists also of N-terminal truncated species (Aβn-40/42 where *n* = 2 to 11) of which pyroglutamate-modified Aβ peptides are predominant components. Most N-truncated Aβ are considered to be the degradation products of full-length Aβ although Aβ11–40/42 may be generated intracellularly directly from APP by BACE proteolysis occurring in trans-Golgi network (Perez-Garmendia et al., 2014). AβN3(pE), Aβ peptide bearing amino-terminal pyroglutamate at position 3, has been shown to be a major N-truncated/modified constituent of intracellular, extracellular and vascular Aβ deposits in AD brain tissue (Saido et al., 1995) which progressively accumulates in the brain and could predate the development of AD symptoms. These truncated species are believed to constitute a potential seeding specie in the formation of pathological amyloid aggregates (Saido et al., 1995; Perez-Garmendia et al., 2014) Although we did not specifically assay for these pyroglutamate-modified Aβ peptides, they may have contributed to the intraneuronal and vascular amyloid which was detected by the immunolabeling of total Aß (4G8) in the current study.

### Sub-regional variation in hippocampal amyloid accumulation

Across the sub-regions of the hippocampal formation and entorhinal cortex, amyloid deposition was significantly higher in the CA1, subiculum and entorhinal cortex compared to the CA2 and CA3 regions respectively. This differential pattern may be related to the spatial localization and role of these regions in the hippocampal circuitry (Lavenex and Banta Lavenex, 2013), differential susceptibility of these sub-regions to different pathologies (Small et al., 2011) or the temporal evolution and hierarchical progression of cerebral amyloidosis (Thal et al., 2006).

The entorhinal cortex has been described as the gateway into the hippocampal formation whereas the subiculum and CA1 regions constitute the outflow stations (Goldman-Rakic et al., 1984; Suzuki and Amaral, 2004). Alzheimer pathology tends to spread along the hippocampal circuitry (Thal et al., 2002a; Lace et al., 2009) and this may explain the differential susceptibility and high β-amyloid load in these sub-regions. Besides, in the hierarchical evolution and natural history of amyloid and tau pathologies, these sub-regions are affected earlier in the disease course, compared to other regions such as CA2 and CA3 (Thal et al., 2002b; Lace et al., 2009). The later effect in the CA2 region, in particular may reflect the natural course of disease or the existence of some underlying protective mechanisms operating in the early stages of disease and only giving way in the advanced stage of the disease (Caruana et al., 2012).

The finding of a relatively higher ratio of Aβ-42 compared to Aβ-40 which further increases with the degree of accumulation of AD pathology is in concordance with previous findings (Aho et al., 2006; Selkoe, 2008) demonstrating the predominance of Aβ-42 over Aβ-40 in brain parenchymal amyloid deposits. We have also previously demonstrated a preponderance of Aβ (42) over Aβ-40 in parenchymal and vascular amyloid deposits in non-human primates including squirrel, rhesus monkeys and aging baboons (Ndung'u et al., 2012).

### Differential amyloid and tau deposition between PSND and PSD

Largely, there were no significant differences in the quantity of amyloid and tau deposited across hippocampal regions and markers in PSND compared to PSD groups. This suggests age-related deposition of amyloid and tau in post-stroke survivors. However, total Aβ immunoreactivity was unexpectedly higher in the entorhinal cortex of PSND than PSD. Though not statistically significant, a similar pattern was observed with Aβ-42 in the subiculum, Aβ-40 in the entorhinal cortex, and soluble Aβ in the CA1 sub-region. This suggests that diffuse early amyloid accumulation in post-stroke subjects alone does not explain why some stroke survivors become demented while others remain cognitively intact. Cognitively normal elderly subjects with huge quantities of amyloid pathology but preserved cognitive functioning have been described in the literature (Bennett et al., 2006; Chetelat et al., 2010). It may also imply that amyloid needs the synergy of other pathologies including tau pathology, vascular lesions, brain atrophy, white matter pathology, MTLA in order to produce significant cognitive decline and dementia (Mormino et al., 2009). In a PIB-PET study of elderly subjects-normal, mild cognitive impairment (MCI) and AD, the investigators found that whereas amyloid load (PIB index) and hippocampal atrophy both predicted loss of episodic memory, amyloid deposition alone in the absence of hippocampal atrophy failed to predict episodic memory loss (Mormino et al., 2009). In addition, a recent study by Wong et al. (2016), found that amyloid retention measured by 11C-PiB PET showed no association with cognitive impairment and clusters of neuropsychiatric symptoms suggesting that other plausible biological pathways could be advanced to explain the development and progression of cognitive impairment and neuropsychiatric symptoms following stroke (Wong et al., 2016). A complementary study of our current cohort (Gemmell et al., 2012) found that whereas pyramidal neuronal volume was preserved in the CA regions and entorhinal cortex of the control and PSND groups, subjects in all the demented groups (including PSD) had significant atrophy of these neurons. This would, therefore, suggest that high amyloid load in the PSND group was insufficient to produce dementia because of preserved neuronal volume. This, indeed, may be a signature of brain/cognitive reserve, preserved synaptic integrity or some other compensatory mechanisms (Stern, 2009; Boyle et al., 2013a).

The observation of similarity between amyloid load in the PSD compared to the control group could imply that given similar quantities of amyloid pathology with respect to the controls, the PSD group was demented possibly because of the presence of additional cerebrovascular lesions which lowered the pathological threshold (Snowdon et al., 1997; Esiri et al., 1999); the presence of neuronal atrophy (Gemmell et al., 2012) or the slightly higher and more advanced hyperphosphorylated tau pathology in the CA2 and CA3 sub-regions of the PSD group. A recent study in which total and phosphorylated tau proteins were quantified in the frontal and temporal cortices of subjects with vascular dementia found a selective loss of total tau protein in VaD compared with controls and AD, whereas phosphorylated tau levels were similar to controls in VaD in both regions, but they were increased in the temporal lobes of patients with AD (Mukaetova-Ladinska et al., 2015). These results demonstrated that breaches of microvascular or microstructural tissue integrity subsequent to ischemic injury in older age may modify tau protein metabolism or phosphorylation and have effects on the burden of neurofibrillary pathology (Mukaetova-Ladinska et al., 2015).

### *APOE ε4* genotype and accumulation of amyloid and tau pathologies

Further analysis of the post-stroke cohort suggested that the presence of the *APOE* ε*4* allele was responsible for driving amyloid and tau accumulation in those who possessed the allele (which was present in 50%) of the PSND subjects. Despite the limited size of the sub-cohort, the amyloid load was significantly higher in the PSND group than the PSD group. *APOE* ε*4* has been associated with accumulation of amyloid and/or tau pathology (Nagy et al., 1995; Saito et al., 2002) in AD but the relationship with post-stroke dementia has been conflicting and less well defined. Previous studies in the Newcastle cohort failed to establish a relationship between *APOE* ε4 with post-stroke cognitive impairment at 3 months after the stroke (Rowan et al., 2005) but predicted decline at 1-year follow up (Ballard et al., 2004). Furthermore, these results are in concordance with a previous study examining the genetic associations of vascular dementia subtypes in which an association was found between *APOE*-ε*4* allele and mixed dementia, stroke-related dementia and subcortical ischemic vascular dementia (SIVD) as well as higher Aβ-(42) levels (Jones et al., 2011). Other studies had also reported both positive (Packard et al., 2007; Liu et al., 2012) and negative associations (Gdovinova et al., 2006) although these were largely clinical studies. It is plausible that the *APOE* ε4 allele might have contributed to the higher quantity of amyloid in subregions of the PSND group compared to the PSD group, but further research is required to explore this relationship further.

### Correlation of cognitive scores with AD pathology

In the post-stroke cohort with available cognitive scores, very limited correlation of tau pathology with CAMCOG memory score was established. In this cohort, there was dissociation of cognitive performance and hippocampal Alzheimer pathologic burden. The implication of this may be that AD pathology probably does not contribute very strongly to the substrates of dementia after a stroke event as previously hypothesized (Henon et al., 1997). And if there was any contribution at all, tau pathology probably contributed more than amyloid pathology. Recent reports also suggest that MTLA which had hitherto been widely ascribed to AD pathology, may have a vascular basis (Bastos-Leite et al., 2007; O'Sullivan et al., 2008). The lack of association may also be due to the presence of other pathologies such as synucleinopathies, (the determination of which is beyond the scope of the current study), presence of robust cognitive reserve and other lifestyle and psychosocial factors might also offer reasons for the dissociation between clinical and cognitive measures in the current study. Our study had limitations. We did not quantify specific isoforms of phosphorylated tau protein (R3 or R4) in our cohort as this could yield different amounts in the different subgroups. Alternative techniques including neurochemical approaches such as ELISA could have also be employed to further substantiate the findings from this current study and the potential existence of familywise Type 1 error associated with multiple pairwise comparisons is also acknowledged.

In conclusion, we did not find quantitative AD pathological markers sufficient to separate the non-demented post-stroke from demented post-stroke subjects. Thus, hippocampal AD-pathological mechanisms do not separate non-demented from demented stroke subjects. Neocortical Alzheimer pathology, other non-Alzheimer neurodegenerative pathologies as well as other non-neurodegenerative mechanisms such as vascular and inflammatory/immune mechanisms require further research in order to fully determine the precise pathological substrates of dementia following stroke.

## Author contributions

ROA performed experiments in the study and drafted the first draft of the manuscript. AO provided technical support. LA and AO contributed to critically revising the manuscript for important intellectual content. RK corrected drafts and obtained the funding. All co-authors approved the final version of the manuscript for submission.

### Conflict of interest statement

The authors declare that the research was conducted in the absence of any commercial or financial relationships that could be construed as a potential conflict of interest.

## References

[B1] Aho L. Jolkkonen J. Alafuzoff I. (2006). Beta-amyloid aggregation in human brains with cerebrovascular lesions. Stroke 37, 2940–2945. 10.1161/01.STR.0000248777.44128.93 17095738

[B2] Aizenstein H. J. Nebes R. D. Saxton J. A. Price J. C. Mathis C. A. Tsopelas N. D.. (2008). Frequent amyloid deposition without significant cognitive impairment among the elderly. Arch. Neurol. 65, 1509–1517. 10.1001/archneur.65.11.1509 19001171 PMC2636844

[B3] Akinyemi R. O. Allan L. Owolabi M. O. Akinyemi J. O. Ogbole G. Ajani A.. (2014). Profile and determinants of vascular cognitive impairment in African stroke survivors: the CogFAST Nigeria Study. J. Neurol. Sci. 346, 241–249. 10.1016/j.jns.2014.08.042 25238666

[B4] Akinyemi R. O. Firbank M. Ogbole G. I. Allan L. M. Owolabi M. O. Akinyemi J. O.. (2015). Medial temporal lobe atrophy, white matter hyperintensities and cognitive impairment among Nigerian African stroke survivors. BMC Res. Notes 8, 625. 10.1186/s13104-015-1552-7 26519155 PMC4628353

[B5] Allan L. M. Rowan E. N. Firbank M. J. Thomas A. J. Parry S. W. Polvikoski T. M.. (2011). Long term incidence of dementia, predictors of mortality and pathological diagnosis in older stroke survivors. Brain 134(Pt 12), 3716–3727. 10.1093/brain/awr273 22171356 PMC3235558

[B6] Ballard C. G. Morris C. M. Rao H. O'Brien J. T. Barber R. Stephens S.. (2004). APOE epsilon4 and cognitive decline in older stroke patients with early cognitive impairment. Neurology 63, 1399–1402. 10.1212/01.WNL.0000141851.93193.17 15505155

[B7] Bastos-Leite A. J. van der Flier W. M. van Straaten E. C. Staekenborg S. S. Scheltens P. Barkhof F. (2007). The contribution of medial temporal lobe atrophy and vascular pathology to cognitive impairment in vascular dementia. Stroke 38, 3182–3185. 10.1161/STROKEAHA.107.490102 17962598

[B8] Bennett D. A. Schneider J. A. Arvanitakis Z. Kelly J. F. Aggarwal N. T. Shah R. C.. (2006). Neuropathology of older persons without cognitive impairment from two community-based studies. Neurology 66, 1837–1844. 10.1212/01.wnl.0000219668.47116.e6 16801647

[B9] Boyle P. A. Wilson R. S. Yu L. Barr A. M. Honer W. G. Schneider J. A. (2013a). Much of late life cognitive decline is not due to common neurodegenerative pathologies. Ann. Neurol. 74, 478–489. 10.1002/ana.23964 23798485 PMC3845973

[B10] Boyle P. A. Yu L. Wilson R. S. Schneider J. A. Bennett D. A. (2013b). Relation of neuropathology with cognitive decline among older persons without dementia. Front. Aging Neurosci. 5:50. 10.3389/fnagi.2013.00050 24058343 PMC3766823

[B11] Braak H. Braak E. (1991). Neuropathological stageing of Alzheimer-related changes. Acta Neuropathol. 82, 239–259. 10.1007/BF00308809 1759558

[B12] Caruana D. A. Alexander G. M. Dudek S. M. (2012). New insights into the regulation of synaptic plasticity from an unexpected place: hippocampal area CA2. Learn. Mem. 19, 391–400. 10.1101/lm.025304.111 22904370 PMC3418763

[B13] Chang L. Bakhos L. Wang Z. Venton D. L. Klein W. L. (2003). Femtomole immunodetection of synthetic and endogenous amyloid-beta oligomers and its application to Alzheimer's disease drug candidate screening. J. Mol. Neurosci. 20, 305–313. 10.1385/JMN:20:3:305 14501013

[B14] Chetelat G. Villemagne V. L. Pike K. E. Baron J. C. Bourgeat P. Jones G.. (2010). Larger temporal volume in elderly with high versus low beta-amyloid deposition. Brain 133, 3349–3358. 10.1093/brain/awq187 20739349

[B15] Cordoliani-Mackowiak M. A. Henon H. Pruvo J. P. Pasquier F. Leys D. (2003). Poststroke dementia: influence of hippocampal atrophy. Arch. Neurol. 60, 585–590. 10.1001/archneur.60.4.585 12707073

[B16] de la Torre J. C. Mussivand T. (1993). Can disturbed brain microcirculation cause Alzheimer's disease? Neurol. Res. 15, 146–153. 10.1080/01616412.1993.11740127 8103579

[B17] Esiri M. M. Nagy Z. Smith M. Z. Barnetson L. Smith A. D. (1999). Cerebrovascular disease and threshold for dementia in the early stages of Alzheimer's disease. Lancet 354, 919–920. 10.1016/S0140-6736(99)02355-7 10489957

[B18] Faul F. Erdfelder E. Lang A. G. Buchner A. (2007). G^*^Power 3: a flexible statistical power analysis program for the social, behavioral, and biomedical sciences. Behav. Res. Methods 39, 175–191. 10.3758/BF03193146 17695343

[B19] Firbank M. J. Allan L. M. Burton E. J. Barber R. O'Brien J. T. Kalaria R. N. (2012). Neuroimaging predictors of death and dementia in a cohort of older stroke survivors. J. Neurol. Neurosurg. Psychiatry 83, 263–267. 10.1136/jnnp-2011-300873 22114300 PMC3289833

[B20] Firbank M. J. Burton E. J. Barber R. Stephens S. Kenny R. A. Ballard C.. (2007). Medial temporal atrophy rather than white matter hyperintensities predict cognitive decline in stroke survivors. Neurobiol. Aging 28, 1664–1669. 10.1016/j.neurobiolaging.2006.07.009 16934370

[B21] GBD 2015 Neurological Disorders Collaborator Group (2017). Global, regional, and national burden of neurological disorders during 1990-2015: a systematic analysis for the Global Burden of Disease Study 2015. Lancet Neurol. 16, 877–897. 10.1016/S1474-4422(17)30299-5 28931491 PMC5641502

[B22] Gdovinova Z. Habalova V. Novosadova Z. (2006). Polymorphism of apolipoproteine E in relation with Alzheimer and vascular dementia. Cell Mol. Neurobiol. 26, 1219–1224. 10.1007/s10571-006-9043-y 16758323 PMC11520594

[B23] Gemmell E. Bosomworth H. Allan L. Hall R. Khundakar A. Oakley A. E.. (2012). Hippocampal neuronal atrophy and cognitive function in delayed poststroke and aging-related dementias. Stroke 43, 808–814. 10.1161/STROKEAHA.111.636498 22207507

[B24] Goldman-Rakic P. S. Selemon L. D. Schwartz M. L. (1984). Dual pathways connecting the dorsolateral prefrontal cortex with the hippocampal formation and parahippocampal cortex in the rhesus monkey. Neuroscience 12, 719–743. 10.1016/0306-4522(84)90166-0 6472617

[B25] Henon H. Pasquier F. Durieu I. Godefroy O. Lucas C. Lebert F.. (1997). Preexisting dementia in stroke patients. baseline frequency, associated factors, and outcome. Stroke 28, 2429–2436. 10.1161/01.STR.28.12.2429 9412627

[B26] Henon H. Pasquier F. Durieu I. Pruvo J. P. Leys D. (1998). Medial temporal lobe atrophy in stroke patients: relation to pre-existing dementia. J. Neurol. Neurosurg. Psychiatry 65, 641–647. 10.1136/jnnp.65.5.641 9810931 PMC2170331

[B27] Hixson J. E. Vernier D. T. (1990). Restriction isotyping of human apolipoprotein E by gene amplification and cleavage with HhaI. J. Lipid Res. 31, 545–548. 2341813

[B28] Hyman B. T. Trojanowski J. Q. (1997). Consensus recommendations for the postmortem diagnosis of Alzheimer disease from the national institute on aging and the reagan institute working group on diagnostic criteria for the neuropathological assessment of Alzheimer disease. J. Neuropathol. Exp. Neurol. 56, 1095–1097. 10.1097/00005072-199710000-00002 9329452

[B29] Iadecola C. (2004). Neurovascular regulation in the normal brain and in Alzheimer's disease. Nat. Rev. Neurosci. 5, 347–360. 10.1038/nrn1387 15100718

[B30] Jones E. L. Kalaria R. N. Sharp S. I. O'Brien J. T. Francis P. T. Ballard C. G. (2011). Genetic associations of autopsy-confirmed vascular dementia subtypes. Dement Geriatr. Cogn. Disord. 31, 247–253. 10.1159/000327171 21474934

[B31] Kalaria R. N. (2009). Linking cerebrovascular defense mechanisms in brain ageing and Alzheimer's disease. Neurobiol. Aging 30, 1512–1514. 10.1016/j.neurobiolaging.2007.10.020 18187235

[B32] Kalaria R. N. (2012a). Cerebrovascular disease and mechanisms of cognitive impairment: evidence from clinicopathological studies in humans. Stroke 43, 2526–2534. 10.1161/STROKEAHA.112.655803 22879100

[B33] Kalaria R. N. (2012b). Risk factors and neurodegenerative mechanisms in stroke related dementia. Panminerva Med. 54, 139–148. 22801431

[B34] Kalaria R. N. Akinyemi R. Ihara M. (2012). Does vascular pathology contribute to Alzheimer changes? J. Neurol. Sci. 322, 141–147. 10.1016/j.jns.2012.07.032 22884479

[B35] Kalaria R. N. Akinyemi R. Ihara M. (2016). Stroke injury, cognitive impairment and vascular dementia. Biochim. Biophys. Acta 1862, 915–925. 10.1016/j.bbadis.2016.01.015 26806700 PMC4827373

[B36] Kalaria R. N. Bhatti S. U. Lust W. D. Perry G. (1993a). The amyloid precursor protein in ischemic brain injury and chronic hypoperfusion. Ann. N. Y. Acad. Sci. 695, 190–193. 10.1111/j.1749-6632.1993.tb23050.x 8239281

[B37] Kalaria R. N. Bhatti S. U. Palatinsky E. A. Pennington D. H. Shelton E. R. Chan H. W.. (1993b). Accumulation of the beta amyloid precursor protein at sites of ischemic injury in rat brain. Neuroreport 4, 211–214. 10.1097/00001756-199302000-00025 8453062

[B38] Kalaria R. N. Kenny R. A. Ballard C. G. Perry R. Ince P. Polvikoski T. (2004). Towards defining the neuropathological substrates of vascular dementia. J. Neurol. Sci. 226, 75–80. 10.1016/j.jns.2004.09.019 15537525

[B39] Katzman R. Terry R. DeTeresa R. Brown T. Davies P. Fuld P.. (1988). Clinical, pathological, and neurochemical changes in dementia: a subgroup with preserved mental status and numerous neocortical plaques. Ann. Neurol. 23, 138–144. 10.1002/ana.410230206 2897823

[B40] Kitaguchi H. Tomimoto H. Ihara M. Shibata M. Uemura K. Kalaria R. N.. (2009). Chronic cerebral hypoperfusion accelerates amyloid beta deposition in APPSwInd transgenic mice. Brain Res. 1294, 202–210. 10.1016/j.brainres.2009.07.078 19646974

[B41] Lace G. Savva G. M. Forster G. de Silva R. Brayne C. Matthews F. E.. (2009). Hippocampal tau pathology is related to neuroanatomical connections: an ageing population-based study. Brain 132(Pt 5), 1324–1334. 10.1093/brain/awp059 19321462

[B42] Lavenex P. Banta Lavenex P. (2013). Building hippocampal circuits to learn and remember: insights into the development of human memory. Behav. Brain Res. 254, 8–21. 10.1016/j.bbr.2013.02.007 23428745

[B43] Lewis H. Beher D. Cookson N. Oakley A. Piggott M. Morris C. M.. (2006). Quantification of Alzheimer pathology in ageing and dementia: age-related accumulation of amyloid-beta(42) peptide in vascular dementia. Neuropathol. Appl. Neurobiol. 32, 103–118. 10.1111/j.1365-2990.2006.00696.x 16599940

[B44] Liu X. Li L. Liu F. Deng S. Zhu R. Li Q.. (2012). ApoE gene polymorphism and vascular dementia in Chinese population: a meta-analysis. J. Neural. Transm. 119, 387–394. 10.1007/s00702-011-0714-6 21984189

[B45] Luchsinger J. A. (2010). Diabetes, related conditions, and dementia. J. Neurol. Sci. 299, 35–38. 10.1016/j.jns.2010.08.063 20888602 PMC2993820

[B46] Marchant N. L. Reed B. R. DeCarli C. S. Madison C. M. Weiner M. W. Chui H. C.. (2012). Cerebrovascular disease, beta-amyloid, and cognition in aging. Neurobiol. Aging 33, 1006.e25-100636. 10.1016/j.neurobiolaging.2011.10.001 22048124 PMC3274647

[B47] Mirra S. S. Heyman A. McKeel D. Sumi S. M. Crain B. J. Brownlee L. M.. (1991). The consortium to establish a registry for Alzheimer's Disease (CERAD). part II. Standardization of the neuropathologic assessment of Alzheimer's disease. Neurology 41, 479–486. 10.1212/WNL.41.4.479 2011243

[B48] Mok V. Leung E. Y. Chu W. Chen S. Wong A. Xiong Y.. (2010). Pittsburgh compound B binding in poststroke dementia. J. Neurol. Sci. 290, 135–137. 10.1016/j.jns.2009.12.014 20056250

[B49] Mormino E. C. Kluth J. T. Madison C. M. Rabinovici G. D. Baker S. L. Miller B. L.. (2009). Episodic memory loss is related to hippocampal-mediated beta-amyloid deposition in elderly subjects. Brain 132(Pt 5), 1310–1323. 10.1093/brain/awn320 19042931 PMC2677792

[B50] Mufson E. J. Chen E. Y. Cochran E. J. Beckett L. A. Bennett D. A. Kordower J. H. (1999). Entorhinal cortex beta-amyloid load in individuals with mild cognitive impairment. Exp. Neurol. 158, 469–490. 10.1006/exnr.1999.7086 10415154

[B51] Mukaetova-Ladinska E. B. Abdel-All Z. Mugica E. S. Li M. Craggs L. J. Oakley A. E.. (2015). Tau proteins in the temporal and frontal cortices in patients with vascular dementia. J. Neuropathol. Exp. Neurol. 74, 148–157. 10.1097/NEN.0000000000000157 25575131

[B52] Nagy Z. Esiri M. M. Jobst K. A. Johnston C. Litchfield S. Sim E.. (1995). Influence of the apolipoprotein E genotype on amyloid deposition and neurofibrillary tangle formation in Alzheimer's disease. Neuroscience 69, 757–761. 10.1016/0306-4522(95)00331-C 8596645

[B53] Ndung'u M. Hartig W. Wegner F. Mwenda J. M. Low R. W. Akinyemi R. O.. (2012). Cerebral amyloid beta(42) deposits and microvascular pathology in ageing baboons. Neuropathol. Appl. Neurobiol. 38, 487–499. 10.1111/j.1365-2990.2011.01246.x 22126319

[B54] Nelson P. T. Alafuzoff I. Bigio E. H. Bouras C. Braak H. Cairns N. J.. (2012). Correlation of Alzheimer disease neuropathologic changes with cognitive status: a review of the literature. J. Neuropathol. Exp. Neurol. 71, 362–381. 10.1097/NEN.0b013e31825018f7 22487856 PMC3560290

[B55] Nelson P. T. Braak H. Markesbery W. R. (2009). Neuropathology and cognitive impairment in Alzheimer disease: a complex but coherent relationship. J. Neuropathol. Exp. Neurol. 68, 1–14. 10.1097/NEN.0b013e3181919a48 19104448 PMC2692822

[B56] O'Sullivan M. Jouvent E. Saemann P. G. Mangin J. F. Viswanathan A. Gschwendtner A.. (2008). Measurement of brain atrophy in subcortical vascular disease: a comparison of different approaches and the impact of ischaemic lesions. Neuroimage 43, 312–320. 10.1016/j.neuroimage.2008.07.049 18722537

[B57] Packard C. J. Westendorp R. G. Stott D. J. Caslake M. J. Murray H. M. Shepherd J.. (2007). Association between apolipoprotein E4 and cognitive decline in elderly adults. J. Am. Geriatr. Soc. 55, 1777–1785. 10.1111/j.1532-5415.2007.01415.x 17979899

[B58] Perez-Garmendia R. Hernandez-Zimbron L. F. Morales M. A. Luna-Munoz J. Mena R. Nava-Catorce M.. (2014). Identification of N-terminally truncated pyroglutamate amyloid-beta in cholesterol-enriched diet-fed rabbit and AD brain. J. Alzheimers Dis. 39, 441–455. 10.3233/JAD-130590 24240639

[B59] Perry R. H. Oakley A. (1993). Newcastle Brain Map. London: Wolfe.

[B60] Petrovitch H. Ross G. W. Steinhorn S. C. Abbott R. D. Markesbery W. Davis D.. (2005). AD lesions and infarcts in demented and non-demented Japanese-American men. Ann. Neurol. 57, 98–103. 10.1002/ana.20318 15562458

[B61] Petrovitch H. White L. R. Izmirilian G. Ross G. W. Havlik R. J. Markesbery W.. (2000). Midlife blood pressure and neuritic plaques, neurofibrillary tangles, and brain weight at death: the HAAS. Honolulu-Asia aging study. Neurobiol. Aging 21, 57–62. 10.1016/S0197-4580(00)00106-8 10794849

[B62] Rogers I. Kerr F. Martinez P. Hardy J. Lovestone S. Partridge L. (2012). Ageing increases vulnerability to abeta42 toxicity in Drosophila. PLoS ONE 7:e40569. 10.1371/journal.pone.0040569 22808195 PMC3395685

[B63] Rowan E. Morris C. M. Stephens S. Ballard C. Dickinson H. Rao H.. (2005). Impact of hypertension and apolipoprotein E4 on poststroke cognition in subjects >75 years of age. Stroke 36, 1864–1868. 10.1161/01.STR.0000177524.17424.2a 16051894

[B64] Saido T. C. Iwatsubo T. Mann D. M. Shimada H. Ihara Y. Kawashima S. (1995). Dominant and differential deposition of distinct beta-amyloid peptide species, A beta N3(pE), in senile plaques. Neuron 14, 457–466. 10.1016/0896-6273(95)90301-1 7857653

[B65] Saito Y. Suzuki K. Nanba E. Yamamoto T. Ohno K. Murayama S. (2002). Niemann-pick type C disease: accelerated neurofibrillary tangle formation and amyloid beta deposition associated with apolipoprotein E epsilon 4 homozygosity. Ann. Neurol. 52, 351–355. 10.1002/ana.10266 12205649

[B66] Schneider J. A. Wilson R. S. Bienias J. L. Evans D. A. Bennett D. A. (2004). Cerebral infarctions and the likelihood of dementia from Alzheimer disease pathology. Neurology 62, 1148–1155. 10.1212/01.WNL.0000118211.78503.F5 15079015

[B67] Selkoe D. J. (2008). Biochemistry and molecular biology of amyloid beta-protein and the mechanism of Alzheimer's disease. Handb. Clin. Neurol. 89, 245–260. 10.1016/S0072-9752(07)01223-7 18631749

[B68] Small S. A. Schobel S. A. Buxton R. B. Witter M. P. Barnes C. A. (2011). A pathophysiological framework of hippocampal dysfunction in ageing and disease. Nat. Rev. Neurosci. 12, 585–601. 10.1038/nrn3085 21897434 PMC3312472

[B69] Snowdon D. A. Greiner L. H. Mortimer J. A. Riley K. P. Greiner P. A. Markesbery W. R. (1997). Brain infarction and the clinical expression of Alzheimer disease. the nun study. JAMA 277, 813–817. 10.1001/jama.1997.03540340047031 9052711

[B70] Stern Y. (2009). Cognitive reserve. Neuropsychologia 47, 2015–2028. 10.1016/j.neuropsychologia.2009.03.004 19467352 PMC2739591

[B71] Suzuki W. A. Amaral D. G. (2004). Functional neuroanatomy of the medial temporal lobe memory system. Cortex 40, 220–222. 10.1016/S0010-9452(08)70958-4 15070014

[B72] Thal D. R. Capetillo-Zarate E. Del Tredici K. Braak H. (2006). The development of amyloid beta protein deposits in the aged brain. Sci. Aging Knowledge Environ. 2006:re1. 10.1126/sageke.2006.6.re1 16525193

[B73] Thal D. R. Ghebremedhin E. Rub U. Yamaguchi H. Del Tredici K. Braak H. (2002a). Two types of sporadic cerebral amyloid angiopathy. J. Neuropathol. Exp. Neurol. 61, 282–293. 10.1093/jnen/61.3.282 11895043

[B74] Thal D. R. Rub U. Orantes M. Braak H. (2002b). Phases of A beta-deposition in the human brain and its relevance for the development of AD. Neurology 58, 1791–1800. 10.1212/WNL.58.12.1791 12084879

[B75] Tomlinson B. E. Blessed G. Roth M. (1968). Observations on the brains of non-demented old people. J. Neurol. Sci. 7, 331–356. 10.1016/0022-510X(68)90154-8 5707082

[B76] Whitehead S. N. Hachinski V. C. Cechetto D. F. (2005). Interaction between a rat model of cerebral ischemia and beta-amyloid toxicity: inflammatory responses. Stroke 36, 107–112. 10.1161/01.STR.0000149627.30763.f9 15591213

[B77] Wong A. Lau A. Y. Yang J. Wang Z. Liu W. Lam B. Y.. (2016). Neuropsychiatric symptom clusters in stroke and transient ischemic attack by cognitive status and stroke subtype: frequency and relationships with vascular lesions, brain atrophy and amyloid. PLoS ONE 11:e0162846. 10.1371/journal.pone.0162846 27632159 PMC5025073

[B78] Writing Group Members Mozaffarian D. Benjamin E. J. Go A. S. Arnett D. K. Blaha M. J.. (2016). Executive summary: heart disease and stroke statistics– update: a report from the American heart association. Circulation 133, 447–454. 10.1161/CIR.0000000000000366 26811276

[B79] Yamada M. Ihara M. Okamoto Y. Maki T. Washida K. Kitamura A.. (2011). The influence of chronic cerebral hypoperfusion on cognitive function and amyloid beta metabolism in APP overexpressing mice. PLoS ONE 6:e16567. 10.1371/journal.pone.0016567 21305033 PMC3029398

